# Editorial: Molecular physiology of invertebrate digestive system

**DOI:** 10.3389/fphys.2023.1304915

**Published:** 2023-10-11

**Authors:** Alexandre Lobo-da-Cunha, Morena Casartelli, Gianluca Tettamanti

**Affiliations:** ^1^ Department of Microscopy, Institute of Biomedical Sciences Abel Salazar (ICBAS), University of Porto, Porto, Portugal; ^2^ Department of Biosciences, University of Milano, Milano, Italy; ^3^ Department of Biotechnology and Life Sciences, University of Insubria, Varese, Italy

**Keywords:** insects, extracellular digestion, intracellular digestion, gut microbiota, digestive metabolism

Invertebrate animals occur in all aquatic and terrestrial habitats from deep-sea to deserts, far outnumbering vertebrates and exceeding them in biological diversity, being essential for ecosystem functioning and services (Laumer et al., 2019; Eisenhauer and Hines, 2021). Invertebrates range in size from microscopic rotifers to giant squids, feed on a huge variety of food sources, and some evolved to become efficient parasites. Their digestive systems reflect this diversity. Animal wellbeing and performance rely on organic nutrients, with the digestive system playing a pivotal role in processing food and providing the body with molecules. These molecules are either utilized for energy or serve as essential substrates for various biosynthetic processes (Karasov and Douglas, 2013). Nevertheless, this is not true for all metazoans because some invertebrates lack a digestive system. In these cases, the digestive system is functionally replaced by other structures [e.g., the integument in parasitic worms (Dalton et al., 2004)], or by symbiotic chemoautotrophy microorganisms which supply nutrients to their host as in the case of adults of deep-sea hydrothermal vent tubeworms (Bright and Lallier, 2010). Other invertebrates lacking the digestive system can be found among sponges, placozoans, and bivalves (Gustafson and Reid, 1988). For example, most sponges simply capture microscopic organic particles suspended in water by phagocytosis or endocytosis and digest them into choanocytes, relying entirely on intracellular digestive processes. In placozoans, the lipophilic cells of the ventral epithelium secrete digestive enzymes over algae and then the absorptive cells collect products of external digestion by endocytosis for further breakdown in lysosomes. A true digestive compartment can be found for the first time in cnidarians, which have a simple digestive system consisting of a single opening that leads to a gastrovascular cavity where extracellular digestion takes place by action of secreted enzymes (Steinmetz, 2019).

Much more complex digestive systems consisting of a tubular digestive tract associated with glands that secrete enzymes for extracellular digestion can be found in Bilateria. Among invertebrates, the most elaborate digestive systems with complex mouthparts adapted to a large variety of feeding strategies can be observed in molluscs and arthropods. In molluscs, although extracellular digestion and absorption can start in the foregut, most of digestion, absorption, and storage of reserves occur in the diverticula of the digestive gland. In this organ, basophilic cells are specialized in secretion of enzymes for extracellular digestion, while digestive cells collect products of extracellular digestion by endocytosis to complete the digestion in lysosomes (Lobo-da-Cunha, 2019). In general, arthropods and other ecdysozoans rely mostly on extracellular digestion to obtain simple molecules that are transported through the absorbing epithelial cells which are characterized by a dense apical brush border (Steinmetz, 2019). In decapod crustaceans the hepatopancreas is the major digestive organ, with F cells responsible for secretion of digestive enzymes and R cells that absorb nutrients. The precise function of B cells, another cell type within this organ, remains unclear. There has been a hypothesis suggesting that these cells might play a role in lipid digestion (Vogt, 2019). In insects, the largest and most diverse group of organisms on Earth which has been able to exploit an extraordinary range of ecological niches and diets, the digestive system basically consists of a tube running from the mouth to the anus, but remarkable morphofunctional differentiation can be observed among different species according to the nutritional quality and the texture of the food (Dow, 1986). The alimentary canal is organized in three main regions with different features, functions, and embryonic origin (i.e., the foregut, the midgut, and the hindgut), where the midgut carries out food digestion and nutrient absorption. In particular, columnar cells, the predominant cell type in this gut region, are responsible for digestive enzyme production and absorption of nutrients (Caccia et al., 2019). The microbiota associated to the insect gut has an important impact on the physiology of these organisms as it contributes to the host fitness by supplying essential nutrients, supporting food digestion and degradation of ingested toxic molecules, and modulating defense mechanisms (Engel and Moran, 2013; Douglas, 2015; Jing et al., 2020).

In insects and other invertebrates, the digestive system serves not only for the digestion and absorption of nutrients but also plays a role in defense against pathogens (Zeng et al., 2022). Additionally, it is equipped with enteroendocrine and nervous cells that participate in regulating feeding and digestion, as well as modulating communication between the alimentary canal and other organs (Wegener and Veenstra, 2015; García-Arrarás et al., 2019; Tierney, 2020).

Despite a wide literature on this topic, information is still fragmentary and much remains to be investigated about the physiology of invertebrate digestive systems, especially at cellular and molecular level. Hence, this Research Topic aimed at providing fresh knowledge on the subject. In this scenario, the study of digestive system of insects is of particular importance due to their relevance as pests and disease vectors, but also as pollinators and protein source.

The relevance of the gut microbiota in supporting nutritional requirement of the insect host is the object of two studies in this Research Topic. Qian et al. demonstrated that the microbiota, in particular bacteria belonging to the phylum Saccharibacteria, plays an important role during diapause in the lepidopteran species *Clanis bilineata tsingtauica* as it supplies nutrient and regulates nutrient metabolism, supporting the insect during this peculiar life period. By using a multi-omics approach Zhang et al. investigated how the functionality of gut microbiota facilitates the exploitation of lignocellulose in the larvae of the coleopteran *Apriona swainsoni*, which attack healthy trees and live inside main branches and trunks, causing high mortality in the host plant.

The impact of feeding on native *versus* exotic host plant on different physiological parameters was evaluated in an herbivorous insect, the lepidopteran *Junonia coenia*, by Mo and Smilanich. In particular, the authors examined if and how feeding on different host plants affects immune response, development, and food exploitation.

The study by Van Lommel et al. compared the gene expression profile in the midgut of the desert locust, *Schistocerca gregaria*, at different time points after feeding, shedding light on midgut functionality in this hemimetabolous insect.

Finally, two studies deal with the kissing bug *Rhodnious prolixus*, a primary vector of Chagas disease and a model species for studying insect physiology, especially concerning blood digestion. A lot of information is available in the literature on the mechanisms and regulation of protein digestion in this insect, but carbohydrates digestion is far less studied. Gama et al. examined this aspect, studying the physiological role of fucosidase, one of the major exoglycosidases in *R. prolixus*, and clarifying the mechanism of its production after blood feeding. The second study, by Moraes et al., demonstrated that RNAi silencing of acetyl-CoA carboxylase in the fat body of *R. prolixus* adult females negatively affects digestion, synthesis of lipids, and reproduction. Since the ingested blood contains low amount of lipids, this insect relies on amino acids derived from blood proteins to synthesize lipids. The results of this study unravel a central role of *de novo* lipogenesis, and in particular of acetyl-CoA carboxylase which is the enzyme that catalyzes the first step of *de novo* synthesis of fatty acid, in the fitness of this insect.
